# Down-regulation of ZNF252P-AS1 alleviates ovarian cancer progression by binding miR-324-3p to downregulate LY6K

**DOI:** 10.1186/s13048-021-00933-7

**Published:** 2022-01-03

**Authors:** Li Geng, Zhongqiu Wang, Yongju Tian

**Affiliations:** 1 Department of Pathology, Jinan City People’s Hospital, Shandong First Medical University, Jinan, Shandong 271100 P.R. China; 2 Department of Pediatric Surgery, Jinan City People’s Hospital, Shandong First Medical University, Jinan, Shandong 271100 P.R. China; 3 Department of Gynecology, Yantaishan Hospital, Yantai, Shandong 264001 P.R. China

**Keywords:** Ovarian cancer, ZNF252P-AS1, miR-324-3p, LY6K

## Abstract

**Background:**

Ovarian cancer is a common gynecological malignant disease in women. Our work aimed to study the specific functions of ZNF252P antisense RNA 1 (ZNF252P-AS1) in ovarian cancer.

**Methods:**

ZNF252P-AS1, miR-324-3p, and lymphocyte antigen 6 family member K (LY6K) expression were analyzed by bioinformatics tools in ovarian cancer tissues and was quantified by qRT-PCR in ovarian cancer cells. The effect of ZNF252P-AS1 knockdown, miR-324-3p suppression, and LY6K over-expression on apoptosis, cell viability, invasion, migration, and epithelial to mesenchymal transition (EMT) was determined in vitro by using colony formation and EdU assays, flow cytometry, transwell assay, and Western blot. The interactions between ZNF252P-AS1 and miR-324-3p and between miR-324-3p and LY6K were validated by luciferase assays. The effects of restraining ZNF252P-AS1 in vivo were studied using BALB/c male nude mice.

**Results:**

ZNF252P-AS1 and LY6K levels were up-regulated, while miR-324-3p was declined in ovarian cancer tissues and cells. ZNF252P-AS1 knockdown reduced ovarian cancer cell proliferation, invasion, migration, and EMT, whereas promoted its apoptosis. Besides, ZNF252P-AS1 interacted with miR-324-3p and reversely regulated its level, and miR-324-3p was directly bound to LY6K and negatively regulated its expression. Moreover, ZNF252P-AS1 knockdown reversed the effect of miR-324-3p on cancer cell apoptosis, growth, migration, invasion, and EMT. Similar results were discovered in the rescue experiments between miR-324-3p and LY6K. Additionally, mouse models in vivo experiments further validated that ZNF252P-AS1 knockdown distinctly inhibited tumor growth.

**Conclusion:**

ZNF252P-AS1 mediated miR-324-3p/LY6K signaling to facilitate progression of ovarian cancer.

## Introduction

Ovarian cancer is the second most common malignant tumor in women over 40, especially in developed countries [37]. In terms of all types of cancer, ovarian cancer is the eleventh most common cancer among women, the fifth incentive of cancer-associated deaths in women, and the lethal gynecological cancer [34]. Unfortunately, patients are mostly diagnosed at the advanced stage, and its median survival ratio is less than 15% [5, 28]. Therefore, despite the increased awareness of ovarian cancer, the survival trends have not been significantly changed due to the challenge of early diagnosis. Thus, it is imperative to clarify the molecular mechanisms of this fatal disease.

Long non-coding RNAs (lncRNAs) exerts pivotal functions in cancer development, particularly in the occurrence and metastasis of cancers [23, 39]. In ovarian cancer, dysfunction of several lncRNAs was shown to be related to cancer progression and metastasis, and they usually function as ceRNAs to regulate biological processes of cells or pathways. LncRNA LINC00319 intensified the progression of ovarian cancer via the miR-423-5p/NACC1 signaling [7], lncRNA MALAT1 accelerates the proliferation and metastasis of ovarian cancer through regulating the PI3K/AKT pathway [13], and lncRNA PVT1 facilitates ovarian cancer progression by targeting miR-214 [4], for example. LncRNA ZNF252P antisense RNA 1 (ZNF252P-AS1) is located on chromosome 8, it is a novel identified lncRNA in hepatocellular carcinoma and might be an independent prognostic biomarker for hepatitis B virus-positive hepatocellular carcinoma patients [47]. Besides, ZNF252P-AS1 is associated with hypertensive nephropathy via insulin signaling pathway [40]. However, the roles of lncRNA ZNF252P-AS1 in ovarian cancer have not been studied.

MicroRNAs (miRNAs) are kinds of single stranded non-coding RNA molecules, and their length are approximately 17–25 nucleotides that encoded by the endogenous genes. miRNAs regulate the target gene translation process via combining to the mRNA 3’-UTR of target genes, thereby participating in multiple tumor biological behaviours [42]. Various miRNAs have been identified to be related with ovarian cancer. miR-552 accelerates ovarian cancer progression via PTEN pathway [46]. miR-96 facilitates ovarian cancer malignant progression via targeting FOXO3A [44]. miR-34a reduces cell viability and chemoresistance of ovarian cancer through regulating HDAC1 [25], for instance. In recent years, miR-324-3p is reported to play an important anti-cancer role in many cancers, such as colorectal cancer [9], breast cancer [18], non-small-cell lung cancer [41], and nasopharyngeal carcinoma [20]. There are relatively few research reports on miR-324-3p in ovarian cancer. LncRNA OIP5-AS1 served as an oncogene in ovarian cancer via targeting miR-324-3p [21]. Lymphocyte antigen 6 family member K (LY6K) is located on chromosome 8q24 [38]. High LY6K expression is associated with poor prognosis and survival outcomes in various cancer types, and LY6K inhibition suppresses cell growth of cancers [3]. LY6K is reported to promote glioblastoma tumorigenicity through CAV-1-mediated ERK1/2 pathway enhancement [32]. Moreover, upregulation of the oncogenic LY6K gene contributes bladder cancer development [27]. Yet, the specific functions of LY6K in ovarian cancer is largely unknown.

In this work, we for the first time revealed the expression levels and functions of ZNF252P-AS1 in ovarian cancer and unraveled that ZNF252P-AS1/miR-324-3p/LY6K played significant roles in ovarian cancer.

## Methods

### Bioinformatics analysis

The Cancer Genome Atlas-Ovarian Cancer (TCGA-OV) data analysis of ZNF252P-AS1 level in normal ovary tissues, ovarian cancer tissues, and recurrent ovarian cancer tissues was carried out using GEPIA and R-language tools. The patients were classified into low and high ZNF252P-AS1 level classes according to the median expression levels of ZNF252P-AS1. The relation between ZNF252P-AS1 and ovarian cancer patient survival was developed by Kaplan–Meier plotter database. The log-rank p value, 95% confidence interval (CI) hazard ratio (HR) and median survival were analyzed. The target genes of miR-324-3p were predicted by retrieving the TargetScan and the differentially expressed mRNA screened from TCGA-OV database. Then, the genes were intersected by Venn analysis. Using the TargetScan database, the possible binding sites between ZNF252P-AS1 and miR-324-3p and between miR-324-3p and LY6K were predicted.

### Cell culture and cell transfection

The human normal epithelial cell line of ovary, IOSE80, was obtained from Wuhan Cell Bank (China).The human ovarian cancer cell lines, SKOV3, A2780, HO8910, and OVCAR3 were purchased from ATCC (USA), and the cells were retained with 10% fetal bovine serum added in Dulbecco's modified Eagle Medium (DMEM). All cells were grown in an incubator at 37 °C with 5 percent CO_2_. SKOV3 and A2780 cells were transfected by using Lipofectamine 2000 (Invitrogen, USA) with ZNF252P-AS1 shRNA or empty control shRNA (GenePharma, China) as following the instruction.

### Quantitative Real-Time polymerase chain reaction (qRT-PCR)

The total RNAs from cell lines were extracted using the TRIzol reagent (TIANGEN, China), complete RNAs from cells were collected and cDNA was synthesized using TIANSeq M-MLV (RNase H-) reverse transcriptase (TIANGEN, China). A FastKing One Stage RT-qPCR Kit was used to perform qRT-PCR (TIANGEN, China). The endogenous control genes to normalize the target gene expression were U6 and GAPDH. Primer sequences used were exhibited as follows: U6, forward: 5’-CAGCACATATACTAAAATTGGAACG-3’, reverse: 5’-ACGAATTTGCGTGTCATCC-3’; GAPDH, forward: 5’-TGAATGGGCAGCCGTTAGGA-3’, reverse: 5’-CGCCCAATACGACCAAATCAGAGA-3’; ZNF252P-AS1, forward: 5’-TTCGAGAACCAACCACTCGG-3’, reverse: 5’-CCCACTGTCCCACTGAAGAC-3’; miR-324-3p, forward: 5’-ATCCAGTGCAGGGTCCGAGG-3’, reverse: 5’-GCGGCGGCCCACTGCCCCAGGTG-3’; LY6K, forward: 5’-TGCGAGACAACGAGATCCAG-3’, reverse: 5’-CGGGTCTAGGGGTTGTCAC-3’.

### Colony formation assay

Cells were seeded in 6-well plates and incubated for one week at 37 °C. The cells were then fixed with 4 percent formaldehyde for 10 min and then stained with 0.1% crystal violet dye (Beyotime, Shanghai, China) for 20 min. Colonies (diameter ≥ 100 μm) were counted.

### EdU assay

The cells were seeded on 96-well plates with a density of 1 × 10^5^ cells in each well. EdU solution (Beyotime, China) was diluted with culture medium in a ratio of 1000:1. The amount of 50 μM EdU solution and 100 μM culture medium were applied to each well after transfection and incubated for 2 h. The 4′,6-diamidino-2phenylindole (DAPI, Sigma, USA) was added to well plates to stain the cells. Finally, the results were perceived and photographed under a fluorescence microscope.

### Cell apoptosis

By centrifuging, the cells were (2 × 10^5^ in each well) harvested and were resuspended by 500 μL binding buffer. Then 5 μL of Annexin V-FITC was added to the cells and incubated in the dark for 30 min at 4 °C, then 5 μL of propidium iodide (PI) was added and incubated at room temperature for 5 min. The apoptosis of cells was determined using Annexin-V-FITC Detection Kit (BioVision, USA) through flow cytometry (BD, USA).

### Western blot

Total proteins were collected using RIPA (Beyotime, China) and SDS-PAGE was used to separate them. Samples were transferred to polyvinylidene fluoride (Beyotime, China) membranes and blocked with 5% phosphate buffered saline that containing 5% bovine serum albumin. The primary antibodies used in this experiment are as follows: Bax (1:1000, Abcam, USA), Bcl-2 (1:1000, Abcam, USA), GAPDH (internal control, 1:2000, Abcam, USA), MMP2 (1:1000, Abcam, USA), MMP9 (1:500, Abcam, USA), E-cadherin (1:500, Abcam, USA), N-cadherin (1:1000, Abcam, USA), and vimentin (1:500, Abcam, USA). Membranes were incubated with different primary antibodies at 4 °C overnight. Each membrane was visualized by BeyoECL Plus chemiluminescence solution (Beyotime, China) after incubation with HRP-conjugated secondary antibodies (Beyotime, China).

### Transwell assay

Cell invasion and migration performed using the invasion chambers of Matrigel (BD Biosciences, USA). In brief, the lower chamber used 20 percent fetal bovine serum as the chemoattractant. The invasion cells were fixed with 0.1 percent crystal violet and then stained. Subsequently, a light microscope quantified the cells.

### Luciferase reporter assay

SKOV3 cells and A2780 cells were seeded in 24 well plates with 1 × 10^5^ cells per well. Co-transfection occurred when the cell density was 70–80 percent. Wild type (Wt) or mutant (Mut) ZNF525P-AS1 combined with miR-324-3p mimics or negative control (NC) mimics were co-transfected using Lipofectamine 2000 (Invitrogen, USA). After 48 h, cells were harvested for (Promega) luciferase activity assay as indicated.

### Tumor xenograft

Two groups were allocated to BALB/c male nude mice: the group sh-NC (SKOV3 cells injected) and the sh-ZNF252P-AS1 group (SKOV3 cells injected with ZNF252P-AS1 knockout). Approximately 0.2 mL of cell suspension containing 2 × 10^4^ cells was injected into the right back of each mouse. At the end of the experiment, a digital caliper determined the tumor size of each mouse. Tumor volumes were measured by measuring the length and width every five days. Each mouse was killed 30 days later and tumor tissues were removed for weighting. Animal experiments have been validated and conducted in accordance with institutional guidelines.

### Statistical analysis

GraphPad Prism 8.0 analyzed the data and showed mean ± standard deviation (SD). Student t test or One-Way ANOVA was used to compare the differences between individual groups. Repeated measurements were used to evaluate tumor volumes. Statistical significance was P < 0.05. Each experiment had at least three replications.

## Results

### The ZNF252P-AS1 gene is up-regulated in ovarian cancer

**Fig. 1:**
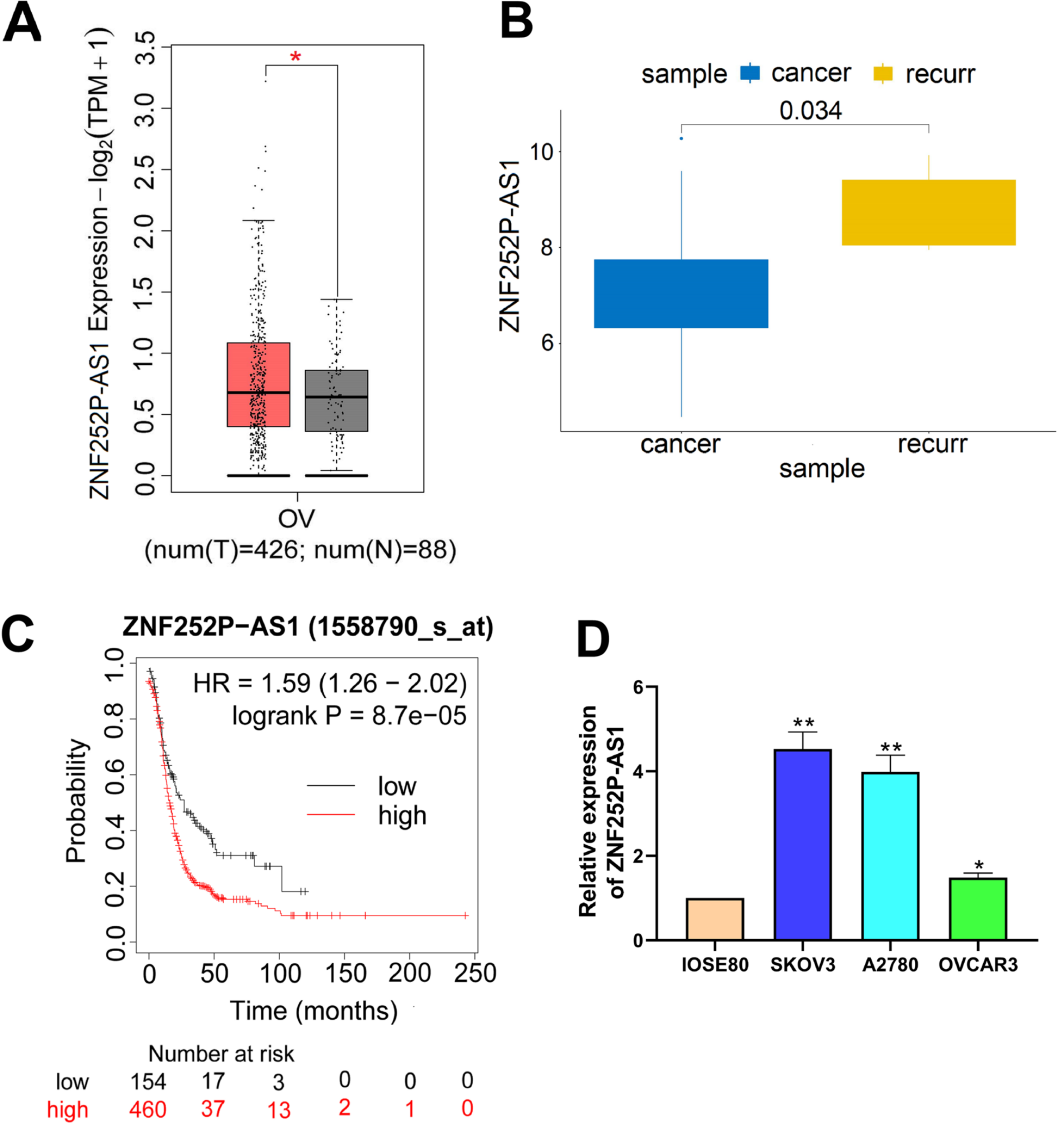
In ovarian cancer, the ZNF252P-AS1 gene is upregulated. A. ZNF252P-AS1 expression levels in normal ovary tissues and ovarian cancer tissues. B. ZNF252P-AS1 expression levels in ovarian cancer tissues and recurrent tissues of ovarian cancer. C. Kaplan–Meier online monitoring of overall survival in ovarian cancer patients with low or high expression of ZNF252P-AS1. D. ZNF252P-AS1 expression was measured by qRT-PCR in IOSE80, SKOV3, A2780, HO8910 and OVCAR3 different cell lines. Data were presented as mean ± SD. *p < 0.05, **p < 0.01.

The expression of the ZNF252P-AS1 gene in normal tissues, ovarian cancer tissues, and frequent ovarian cancer tissues was categorized using TCGA-OV results having 426 ovarian cancer samples and 88 normal controls. The results of the study showed that the expression of ZNF252P-AS1 in ovarian tissues was upregulated relative to normal tissues (Fig. 1A) and that its expression was higher in recurrent ovarian tissues than ovarian tissues (Fig. 1B). The relationship between ZNF252P-AS1 levels and survival in ovarian cancer patients was evaluated by the Kaplan–Meier survival curve. The findings showed that high ZNF252P-AS1 expression was closely linked to poor survival in patients with ovarian cancer (HR = 1.59, 95% CI = 1.26–2.02, Fig. 1C). Afterward, we observed that the expression of ZNF252P-AS1 was distinctly increased in SKOV3, A2780, HO8910 and OVCAR3 ovarian cancer cells compared to IOSE80 normal ovarian cells, and the expression level was highest in SKOV3 and A2780 cells (Fig. 1D). Our findings have shown that lncRNA ZNF252P-AS1 may play a role in ovarian cancer growth.

### Down-regulation of ZNF252P-AS1 inhibits proliferation and promotes apoptosis of ovarian cancer cells

**Fig. 2:**
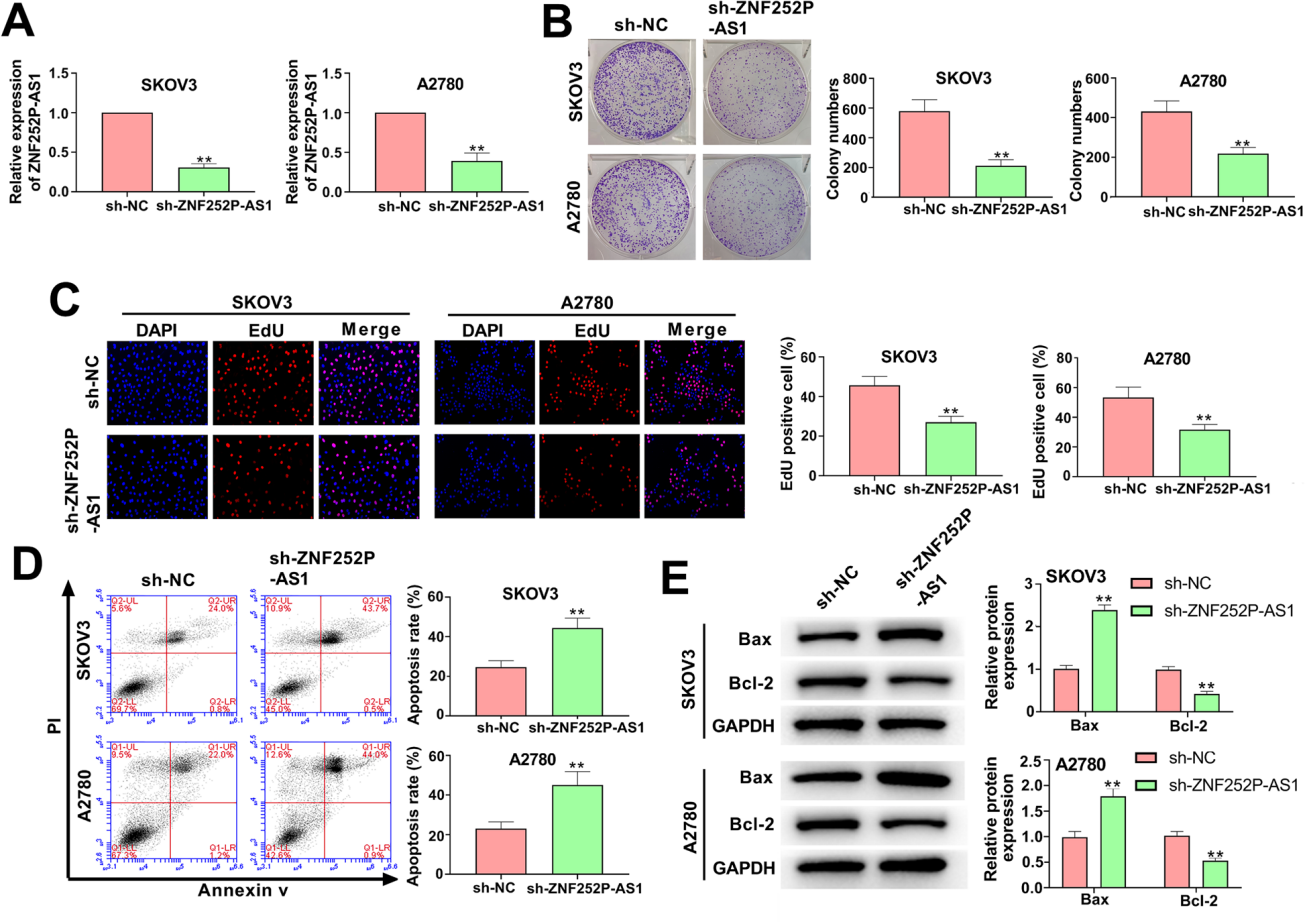
Down-reglulaiotn of ZNF252P-AS1 suppresses proliferation and facilitates apoptosis of ovarian cancer cells. A. Transfection efficiency of sh-ZNF252P-AS1 was confirmed by qRT-PCR in SKOV3 cells and A2780 cells. B. Ovarian cancer cell colony formation was reduced by ZNF252P-AS1 knockdown. C. EdU assays detected the cell viability of SKOV3 and A2780 cells. D. Flow cytometry determined the apoptosis of SKOV3 cells and A2780 cells. E. Western blot detected the expression of Bax and Bcl-2 in SKOV3 and A2780 cells. Data were presented as mean ± SD. **p < 0.01

We reduced the expression of ZNF252P-AS1 in SKOV3 cells and A2780 cells by transferring shRNAs to explore the biological roles of ZNF252P-AS1 in ovarian cancer cells. The qRT-PCR assay verified that ZNF252P-AS1 expression in ovarian cancer cells was significantly downregulated in ovarian cancer cells (Fig. 2A). The colony formation experiment and the EdU experiment indicated that inhibition of ZNF252P-AS1 meaningfully reduced the viability of SKOV3 cells and A2780 cells compared to the conforming controls (Fig. 2B, C). Flow cytometry assay findings showed that suppression of ZNF252P-AS1 in SKOV3 and A2780 cells significantly facilitated cell apoptosis (Fig. 2D). Similarly, the expression of Bax, a pro-apoptotic protein, was remarkably reduced by ZNF252P-AS1 knockdown in SKOV3 and A2780 cells. Conversely, Bcl-2, an anti-apoptotic protein, was significantly increased in ovarian cancer cells when ZNF252P-AS1 was down-regulated (Fig. 2E). These results showed that down-regulation of ZNF252P-AS1 prevented the propagation of ovarian cancer cells and assisted their apoptosis.

### Down-regulation of ZNF252P-AS1 inhibits the migration, invasion, and EMT of ovarian cancer cells

**Fig. 3:**
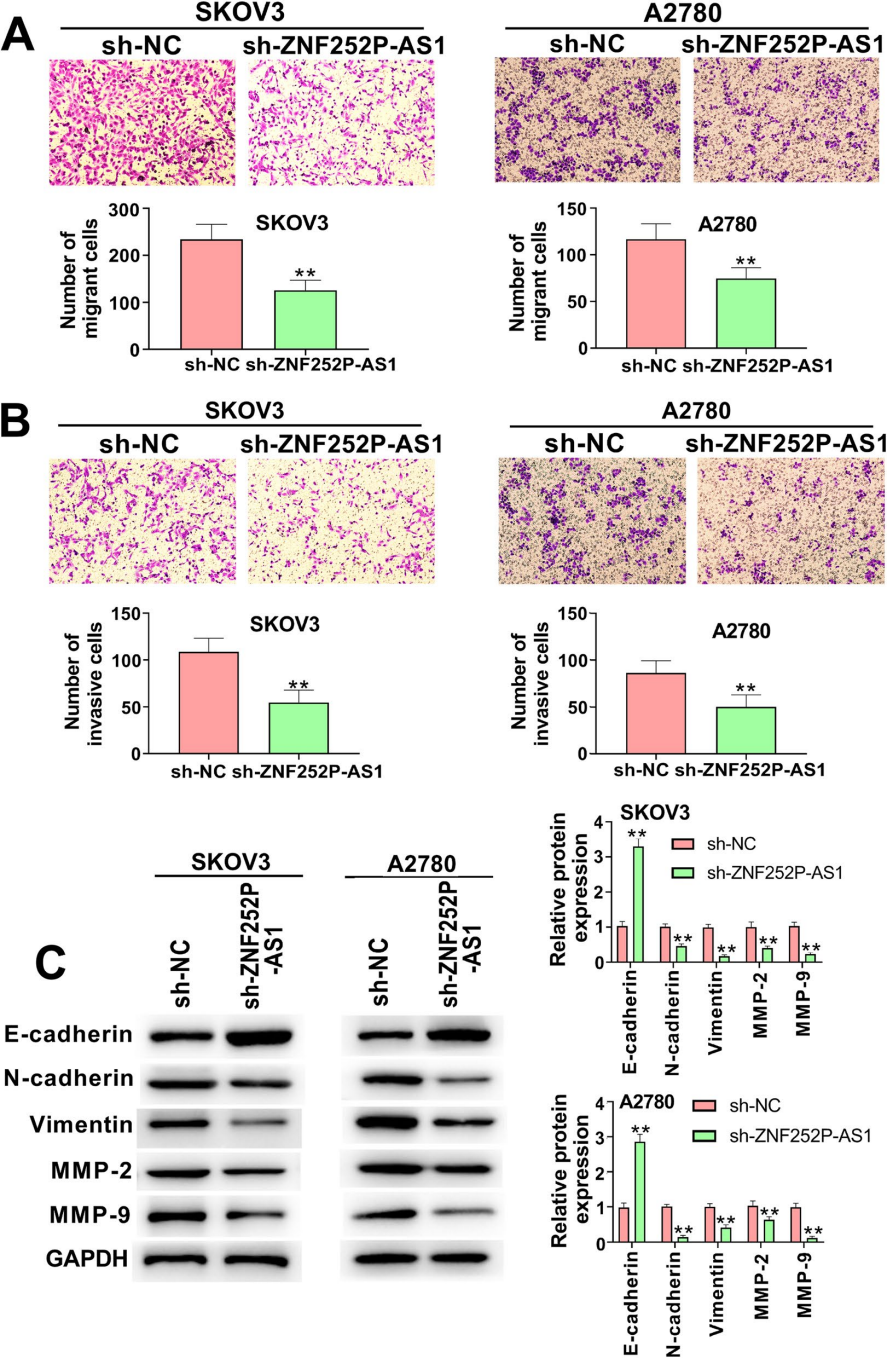
Down-regulation of ZNF252P-AS1 inhibits the migration, invasion, and EMT process of ovarian cancer cells. A. The migrated ability of SKOV3 cells and A2780 cells was measured by transwell assay. B. The invasion of SKOV3 cells and A2780 cells was measured by transwell assay. C. Western blot detected the expression levels of E-cadherin, vimentin, N-cadherin, MMP2, and MMP9 in SKOV3 and A2780 cells. Data were presented as mean ± SD. **p < 0.01

Next, we examined whether ZNF252P-AS1 is active in ovarian cancer metastases. Using the transwell migration assay, we observed that the number of migrating cells in ZNF252P-AS1 knock-down ovarian cancer cells was clearly reduced (Fig. 3A). In addition, the transwell invasion assay revealed that ZNF252P-AS1 suppression greatly decreased the migration capability of SKOV3 cells and A2780 cells (Fig. 3B). Then, we looked for the impact of ZNF252P-AS1 on EMT in ovarian cancer cells. Western blot was used for the measurement of protein expression of EMT-associated proteins such as E-cadherin, vimentin, N-cadherin, MMP2 and MMP9. As seen in Fig. 3.C, the expression of N-cadherin, vimentin, MMP2 and MMP9 was significantly reduced when ZNF252P-AS1 was inhibited in ovarian cancer cells, whereas E-cadherin was dramatically up-regulated. Taken together, our data have shown that the down-regulation of ZNF252P-AS1 prevents the invasion, migration, and EMT mechanism of ovarian cancer cells.

### ZNF252P-AS1 binds to and negatively regulates miR-324-3p

**Fig. 4:**
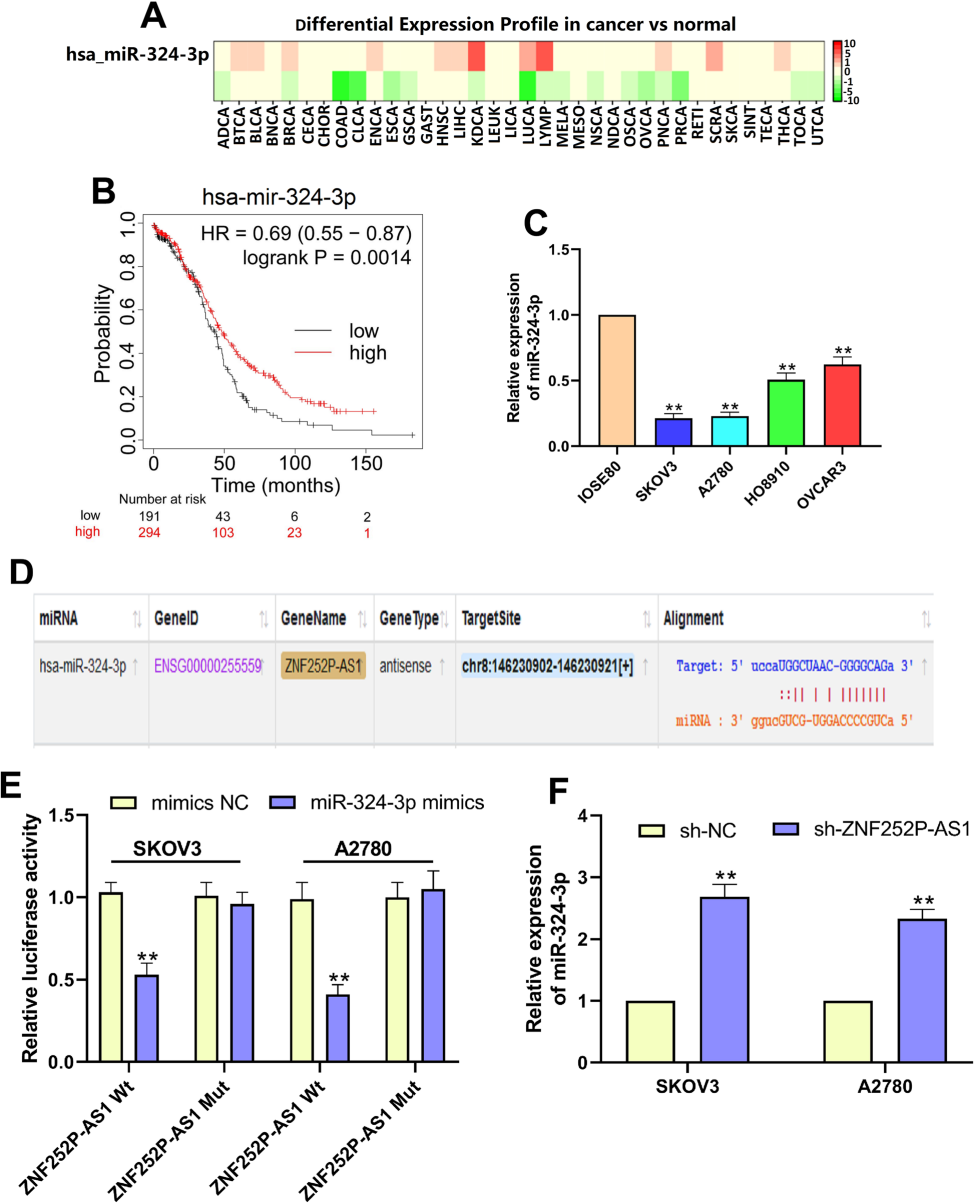
ZNF252P-AS1 binds to and negatively regulates miR-324-3p. A. DbDEMC online tool was used to analyze the differential expression profile of miR-324-3p in various cancers compared to standard controls. B. Kaplan–Meier survival curve for TCGA-OV dataset miR-324-3p level in ovarian cancer tissues. C. qRT-PCR investigated the relative level of miR-324-3p in IOSE80 cells, SKOV3 cells, A2780 cells and OVCAR3 cells. D. Binding sites between ZNF252P-AS1 and miR-324-3p were estimated using the miRDB database. E. A luciferase assay established the binding relationship between ZNF252P-AS1 and miR-324-3p. F. miR-324-3p level was measured by qRT-PCR in SKOV3 cells and A2780 cells. Data were presented as mean ± SD. **p < 0.01

In ovarian cancer, we studied it to identify the regulatory function of ZNF252P-AS1 and found that ZNF252P-AS1 would bind to miR-324-3p. ZNFA252P-AS1 has been broadly expressed in multiple cancer diseases and down-regulated in ovarian cancer, as seen in Fig. 4A.

In comparison, higher miR-324-3p expression was related with higher survival rates for ovarian cancer (Fig. 4.B). First, relative to normal ovarian cells, we studied miR-324-3p expression in ovarian cancer cells. We observed that miR-324-3p in ovarian cancer cells was substantially downregulated and the level of expression was lowest in SKOV3 and A2780 cells (Fig. 4C). The predicted binding sites between ZNF252P-AS1 and miR-324-3p are seen in Fig. 4D. Co-transfection with Wt-ZNF252P-AS1 and miR-324-3p imitate substantially decreased luciferase activity in SKOV3 and A2780 cells. However, when cells were co-transfected with Mut-ZNF252P-AS1 and miR-324-3p mimics, luciferase activity showed no substantial improvement (Fig. 4.E). In SKOV3 cells and A2780 cells, miR-324-3p expression was also greatly improved when ZNF252P-AS1 was inhibited (Fig. 4F). Our data shows that ZNF252-AS1 can bind to miR-324-3p directly and regulate it negatively.

### Down-regulation of ZNF252P-AS1 promotes apoptosis and inhibits proliferation, migration, invasion, and EMT of ovarian cancer through reversely regulating miR-324-3p

**Fig. 5:**
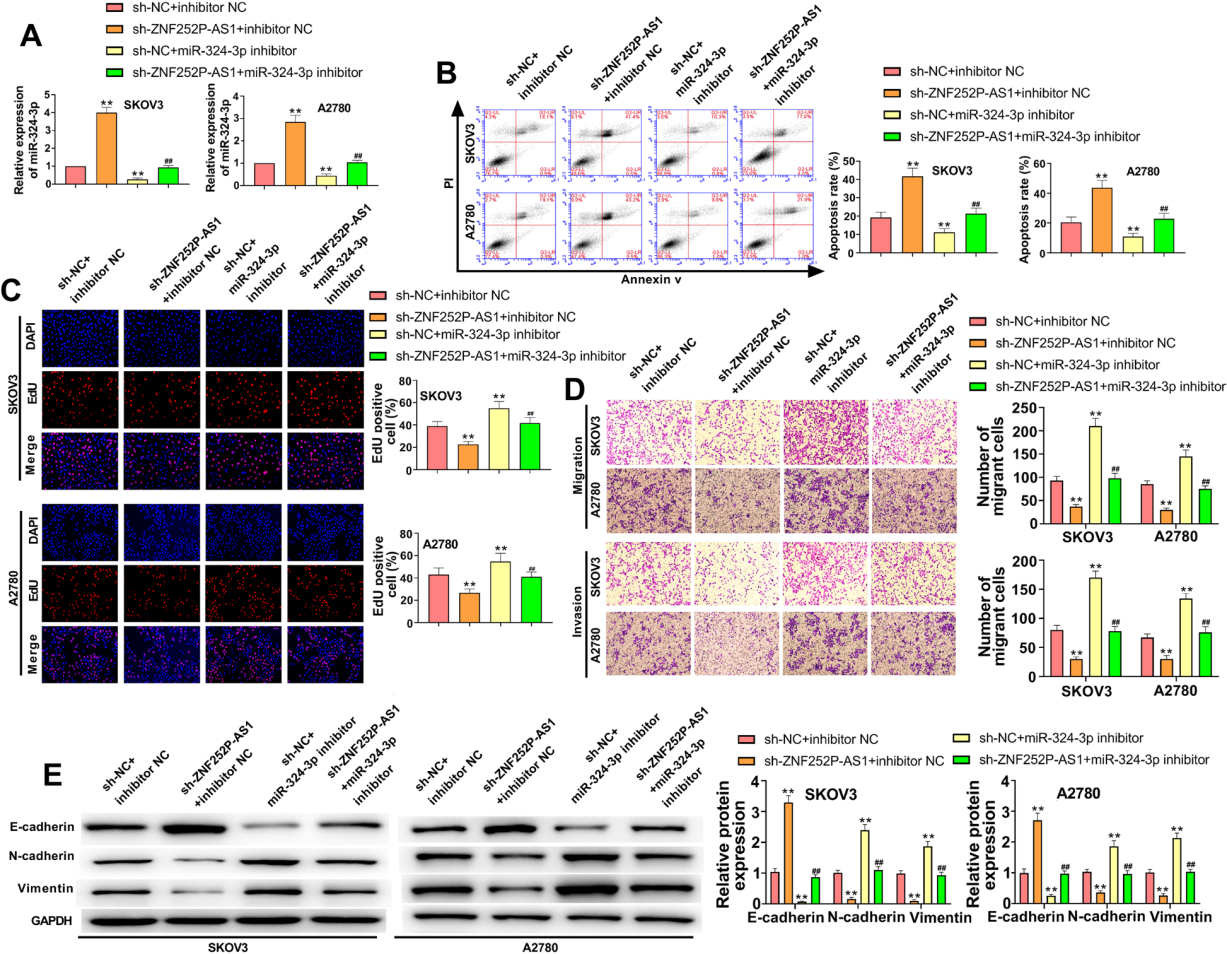
ZNF252P-AS1 knockdown promotes apoptosis and inhibits proliferation, migration, invasion, and EMT of ovarian cancer through reversely regulating miR-324-3p. A. The relative level of miR-324-3p was quantified by qRT-PCR in SKOV3 cells and A2780 cells. B. Flow cytometry was used to determine cell apoptosis for each group. C. Cell growth from each group was measured by the EdU test. D. Transwell assays evaluated the migration and invasion ability of ovarian cancer cells. E. Western blot was used to detect the expression of E-cadherin, vimentin, and N-cadherin in ovarian cancer cells. Data were presented as mean ± SD. **p < 0.01, vs. sh-NC + inhibitor NC. ^##^p < 0.01, vs. sh-ZNF252P-AS1 + inhibitor NC and sh-NC + miR-324-3p inhibitor

Down-regulation of ZNF252P-AS1 led to a significant increase in miR-324-3p expression in ovarian cancer cells, and the transfection of miR-324-3p inhibitor effectively inhibited miR-324-3p expression (Fig. 5A). Flow cytometry assay indicated that miR-324-3p inhibitor led to an important decrease in apoptosis of SKOV3 cells and A2780 cells, whereas the decreased apoptosis was abrogated with the co-transfection of sh-ZNF252P-AS1 and miR-324-3p inhibitor (Fig. 5B). EdU assay revealed that the cell adequacy of ovarian cancer cells was significantly enhanced by miR-324-3p inhibitor, however, the viability of cells was dramatically reduced when the cells were co-transfected with sh-ZNF252P-AS1 and miR-324-5p (Fig. 5C). Transwell assay indicated that miR-324-5p inhibitor promoted the migrated and invaded ability of ovarian cancer cells, while co-transfection of sh-ZNF252P-AS1 and miR-324-3p inhibitor weakened migration and invasion capacity of cells (Fig. 5D). Likewise, as presented in Fig. 5E, miR-324-5p inhibitor dramatically promoted the EMT of ovarian cancer cells, as indicated by the reduced E-cadherin and improved N-cadherin and vimentin. Simultaneously Down-regulating ZNF252P-AS1 and miR-324-5p alleviated the EMT phenotype compared with the corresponding control (Fig. 5E). These results together showed that ZNF252P-AS1 had an effect on the development of ovarian cancer through downewgulating miR-324-3p.

### LY6K is a target of miR-324-3p

**Fig. 6:**
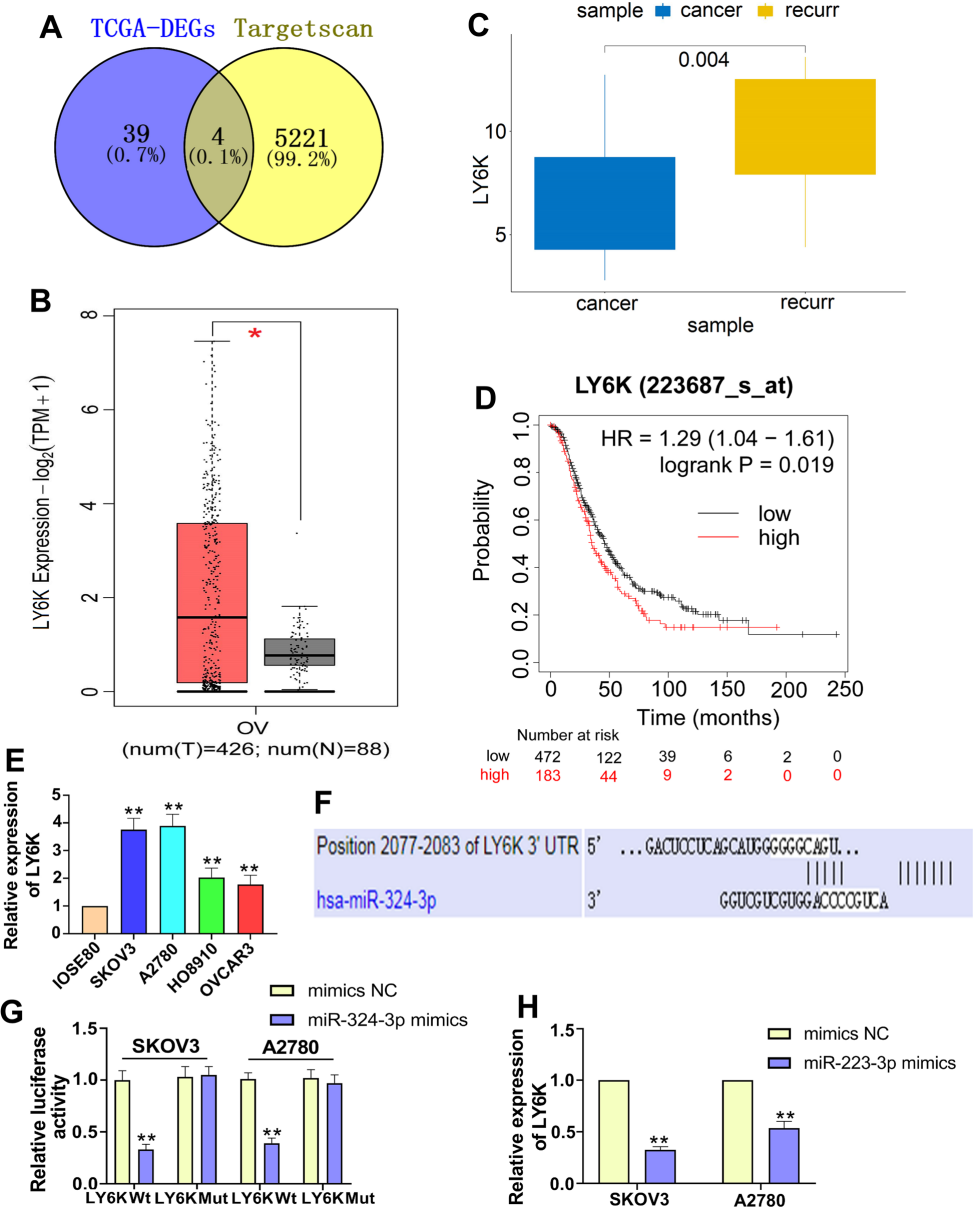
LY6K is a target of miR-324-3p. A. The target genes that may bind to miR-324-3p were predicted by retrieving the TargetScan and the differentially expressed genes screened from TCGA-OV database. B. LY6K mRNA expression levels in normal tissues and ovarian cancer tissues. C. LY6K expression levels in ovarian cancer tissues and recurrent ovarian cancer tissues. D. Online Kaplan–Meier plot of complete survival in ovarian cancer patients with low or high LY6K expression. E. LY6K expression was examined by qRT-PCR in IOSE80, SKOV3, A2780, HO8910, and OVCAR3 cells. F. TargetScan reflected the binding sites between miR-324-3p and LY6K Reporter. G. Luciferase analysis confirmed that LY6K in SKOV3 and A2780 cells was targeted by miR-324-3p. H. After transfection with mimics, qRT-PCR detected the relative expression of LY6K in ovarian cancer cells. Data were presented as mean ± SD. *p < 0.05, **p < 0.01

By searching the TargetScan and the differentially expressed genes screened from TCGA-OV database, the Venn diagram of intersected candidate target genes was plotted, and the four differential expressed genes were LY6K, LRRC55, SMTNL1 and ZC3H12A (Fig. 6A). Moreover, we found that LY6K was the most differentially expressed of these differential expressed genes (Fig. 6A). LY6K is reported to be the target gene for miR-324-3p and that LY6K functions in several cancers [15, 19]. Thus, we subsequently studied LY6K expression in ovary cancer tissue in normal tissues, ovary cancer tissues and recurrent ovary cancer tissues with TCGA-OV data. Analyzed findings found that LY6K is higher in ovarian cancer tissues relative to normal controls (Fig. 6B) and is more pronounced in recurring ovarian cancer tissues compared to cancer tissues (Fig. 6C). The Kaplan–Meier postoperative survival curve for ovarian cancer patients and LY6K expression showed that high LY6K levels were related with poor ovarian cancer prognosis (Fig. 6D). Likewise, qRT-PCR results indicated that LY6K is dramatically up-regulated in ovarian cancer cells associated with standard ovarian cells (Fig. 6E). The potential binding sites were shown in Fig. 6F, and the luciferase reporter assay in SKOV3 cells and A2780 cells provided the validation of the target cognation among miR-324-3p and LY6K (Fig. 6G). Furthermore, the level of LY6K was adversely correlated with miR-324-3p (Fig. 6H). Taken together, our results suggested that miR-324-3p targeted and adversely adjusted LY6K in ovarian cancer.

### miR-324-3p over-expression promotes apoptosis and inhibits proliferation, migration, invasion, and EMT of ovarian cancer cells by negatively regulating LY6K

**Fig. 7:**
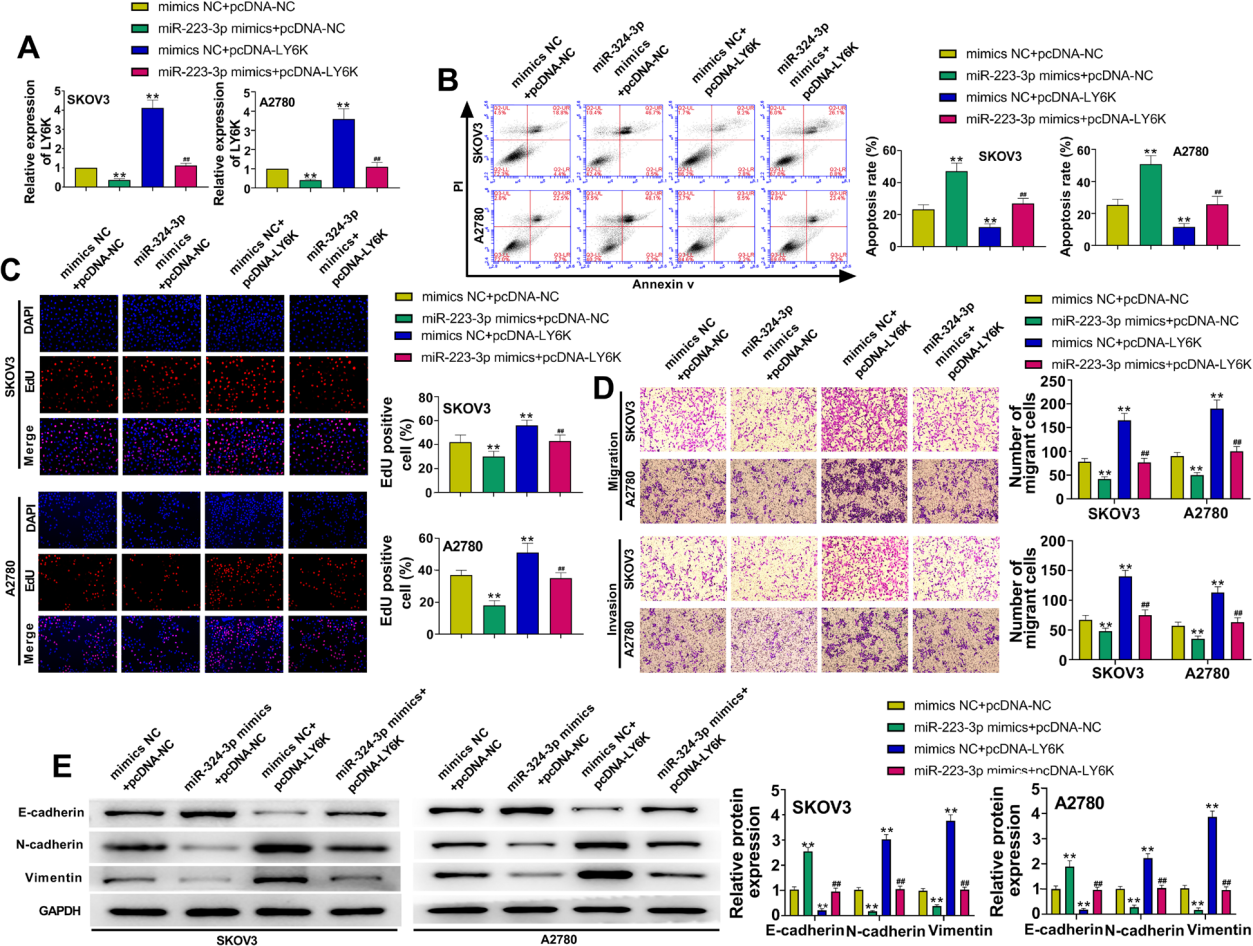
miR-324-3p over-expression promotes apoptosis and inhibits viability, migration, invasion, and EMT of ovarian cancer cells by negatively regulating LY6K. A. After transfection of mimics and plasmids, qRT-PCR observed the relative expression of LY6K in SKOV3 and A2780 cells. B. After transfection of mimics and plasmids, flow cytometry determined cell apoptosis of SKOV3 and A2780 cells. C. Cell proliferation of SKOV3 and A2780 cells after transfection of mimics and plasmids was observed by the EdU assay. D. Transwell assays examined SKOV3 and A2780 cell migration and invasion. E. Western blot was used to identify the expression of E-cadherin, N-cadherin and vimentin in ovarian cancer cells. Data were presented as mean ± SD. **p < 0.01, vs. mimics NC + pcDNA-NC. ^##^p < 0.01, vs. miR-223-3p mimics + pcDNA-NC and mimics NC + pcDNA-LY6K

For further investigating the biological functions of miR-324-3p and LY6K, LY6K was over-expressed and co-transfected with miR-324-3p mimics in SKOV3 cells and A2780 cells. Figure 7.A indicated the confirmation of LY6K expression in ovarian cancer cells. Overexpression of LY6K could refrain the apoptosis of ovarian cancer cells, while overexpressing miR-324-3p and LY6K could alleviate this effect (Fig. 7B). Up-regulation of LY6K significantly enhanced ovarian cancer cell proliferation, while up-regulating miR-324-3p and LY6K neutralized this effect (Fig. 7C). Elevating LY6K expression notably promoted the migration and invasion of cancer cells, however co-transfection of miR-324-3p mimic and pcDNA-LY6K ameliorated the impact (Fig. 7D). Meanwhile, overexpression of miR-324-3p could alleviate the EMT phenotype caused by elevated LY6K (Fig. 7E). Collectively, miR-324-3p over-expression promoted apoptosis and inhibited viability, migration, invasion, and EMT of ovarian cancer cells by negatively regulating LY6K.

### Down-regulation of ZNF252P-AS1 reduces the tumorigenicity of ovarian cancer in vivo

**Fig. 8:**
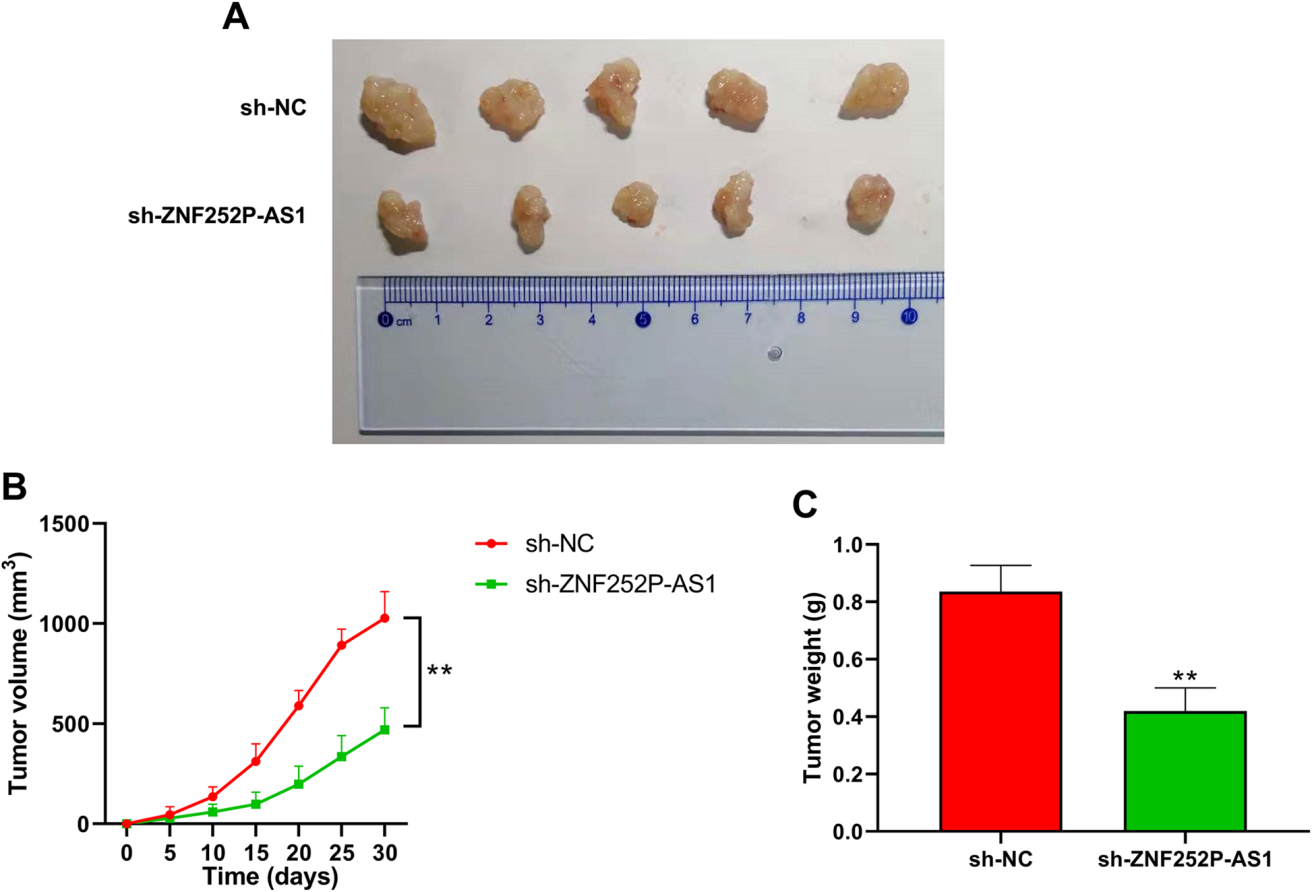
Down-regulation of ZNF252P-AS1 reduces the tumorigenicity of ovarian cancer in vivo. A. Representative tumor size images from nude mice injected with lenti-sh-ZNF252P-AS1 cells injected with SKOV3. B. Tumor volume was measured for each mouse every five days. C. After killing of nude mice, tumor weight of each mouse was evaluated. Data were presented as mean ± SD. **p < 0.01

To examine the conclusion of ZNF252P-AS1 on the tumorigenicity of ovarian cancer, nude mouse xenograft models were constructed. The harvested tumors were depicted in Fig. 8A. Macroscopic observation revealed that ZNF252P-AS1 knockdown could reduce the tumor size in nude mouse models. The tumor volumes were remarkably reduced in ZFN252P-AS1 knockdown mice equated with the sh-NC group (Fig. 8B). Meanwhile, the tumor weight was also markedly decreased by ZNF252P-AS1 knockdown in vivo (Fig. 8C). These results confirmed that down-regulation of ZNF252P-AS1 could reduce the tumorigenicity of ovarian cancer in vivo.

## Discussion

Ovarian cancer is one of the most malignant tumors and known for the highest mortality rate among all gynecological cancers [6, 11, 43]. Only about one-fifth of advanced ovarian cancer patients were effectively cured and survived more than 12 years after treatment [31]. Therefore, the exploration of new drug targets for ovarian cancer diagnosis and treatment is of great significance.

ZNF252P-AS1 has been confirmed to function as an oncogene in hepatocellular carcinoma and hypertensive nephropathy [40, 47]. Similarly, an elevated ZNF252P-AS1 level was found in tissues and cell lines of ovarian cancer in our study, and high ZNF252P-AS1 expression was found in tissues of recurrent ovarian cancer tissues. The prognosis analysis of ZNF252P-AS1 showed that high ZNF252P-AS1 level brought out adverse roles in survival rates according to Kaplan–Meier analysis. Epithelial-mesenchymal transition (EMT) process causes epithelial cells to lose the biological features, whereas obtaining the characteristics of mesenchymal cells. Nevertheless, EMT program can be reactivated by tumor cells, and it will enhance their aggressiveness. Moreover, a number of studies showed that lncRNAs were involved in the process of EMT [33, 45]. In our work, knockdown of ZNF252P-AS1 promoted cell apoptosis and suppressed cell growth, migration capacity, and invasion capacity of ovarian cancer cells. Increasing evidence has demonstrated that EMT plays essential roles in the metastatic dissemination of cancer cells by endowing them with motile and invasive phenotype [26, 48]. In this process, the epithelial marker, such as E-cadherin, is significantly decreased at the molecular level, whereas the mesenchymal markers, such as N-cadherin and vimentin, are dramatically enhanced [22, 29]. To address ZNF252P-AS1 underlying mechanism in the invasiveness of ovarian cancer, the levels of multiple EMT-related factors were detected. The data indicated that ZNF252P-AS1 depletion resulted in an up-regulation of E-cadherin and simultaneous reduction of N-cadherin and vimentin, suggesting that ZNF252P-AS1 might contribute to EMT. A number of evidences illustrated that MMPs are widely known participants in extracellular matrix degradation and rearrangement during the period of cancer cell migration and invasion [1, 30]. MMP2 and MMP9 are the members of matrix metalloproteinases family, which is closely related to poor prognosis in ovarian cancer patients [12, 14, 17]. In the present work, the elimination of ZNF252P-AS1 exhibited decreased expression of MMP2 and MMP9 in ovarian cancer. Collectively, our data illustrated that ZNF252P-AS1 might act as an oncogene in ovarian cancer.

miRNAs exert critical functions in certain physiological processes. In the present work, miR-324-3p level was decreased in ovarian cancer tissues, and high miR-324-3p level was associated with higher survival. We following confirmed the binding between ZNF252P-AS1 and miR-324-3p through a luciferase assay, and miR-324-3p was inversely regulated by ZNF252P-AS1 in ovarian cancer cells. miR-324-3p has disparate biological functions in different human cancers. For example, miR-324-3p accelerates the development of gastric cancer through Smad4-mediated Wnt/β-catenin signaling [35],miR-324-3p restrains the invasion and migration in nasopharyngeal carcinoma via directly targeting WNT2B [20]. LncRNA SNHG22 promotes breast cancer malignant phenotypes by sponging miR-324-3p [10]. Over-expression of miR-324-3p restrains ovarian cancer cell proliferation, invasion, and migration [21]. Likewise, in our study, miR-324-3p served as a tumor suppressor in ovarian cancer. miR-324-3p suppression caused significant increases in cancer cell growth, migration, invasion, and EMT phenotype and obvious reduction in cell apoptosis. However, simultaneous inhibition of ZNF252P-AS1 expression partially alleviated the tumor inhibition effect induced by miR-324-3p inhibitor. Taken together, down-regulation of ZNF252P-AS1 promoted apoptosis and inhibited proliferation, migration, invasion, and EMT of ovarian cancer through reversely regulating miR-324-3p.

High expression of LY6K at mRNA level is reported to be related to poor survival outcomes in various cancers, including breast cancer [15], cervical cancer [36], and colorectal cancer [24]. Previous studies also shows that LY6K is a crucial contributor to tumor biology and functions in glioblastoma tumorigenicity [32], and knockdown of LY6K in cancer cells leads to reduced tumor growth in mouse models [2]. In this study, LY6K expression was lifted in the tissues of ovarian cancer patients, and higher level of LY6K was found in recurrent cancer patients. Besides, a higher expression of LY6K was closely related to poor prognosis in ovarian cancer patients, which is consistent with previous reports. We validated that in ovarian cancer cells miR-324-3p directly targeted and negatively regulated LY6K. Prior studies shows that LY6K expression in normal cells mainly exists in testicular germ cells and functions in the migration of sperm cells [8], miR-500a-3p targets LY6K to suppress cell growth and cell invasion in human non-small lung cancer [19], and the expression of LY6K is upregulated by AP-1 transcription factor to facilitate cell growth and metastatic ability in breast cancer [16]. Nevertheless, there are few reports to elucidate the effect of LY6K in ovarian cancer. In the current work, we found that over-expression of LY6K accelerated ovarian cancer cell growth, invasion, migration, and EMT process, whereas restrained cancer cell apoptosis. However, up-regulation of miR-324-3p neutralized the cancer-promoting effect induced by LY6K overexpression. Our work revealed that ZNF252P-AS1 could promote the progression of ovarian cancer cells at least in part via sponging miR-324-3p to upregulate LY6K.

Additionally, knockdown of ZNF252P-AS1 inhibited ovarian cancer tumor growth in vivo, as indicated by the decreased tumor volume and tumor weight, which further indicated that ZNF252P-AS1 might act as a promoter in ovarian cancer progression. However, the underlying mechanisms of ZNF252P-AS1 in vivo have not yet been fully illustrated. Currently, we conducted in vivo experiments and confirmed the anti-tumor effect of ZNF252P-AS1 knockdown in ovarian cancer. More work is required to investigate how ZNF252P-AS1 can be used as a novel therapeutic target for ovarian cancer treatment and to design more efficient treatment regimens. Pharmacological inhibition of ZNF252P-AS1 may be choosed for cancer treatment in the future.

## Conclusions

**Fig. 9:**
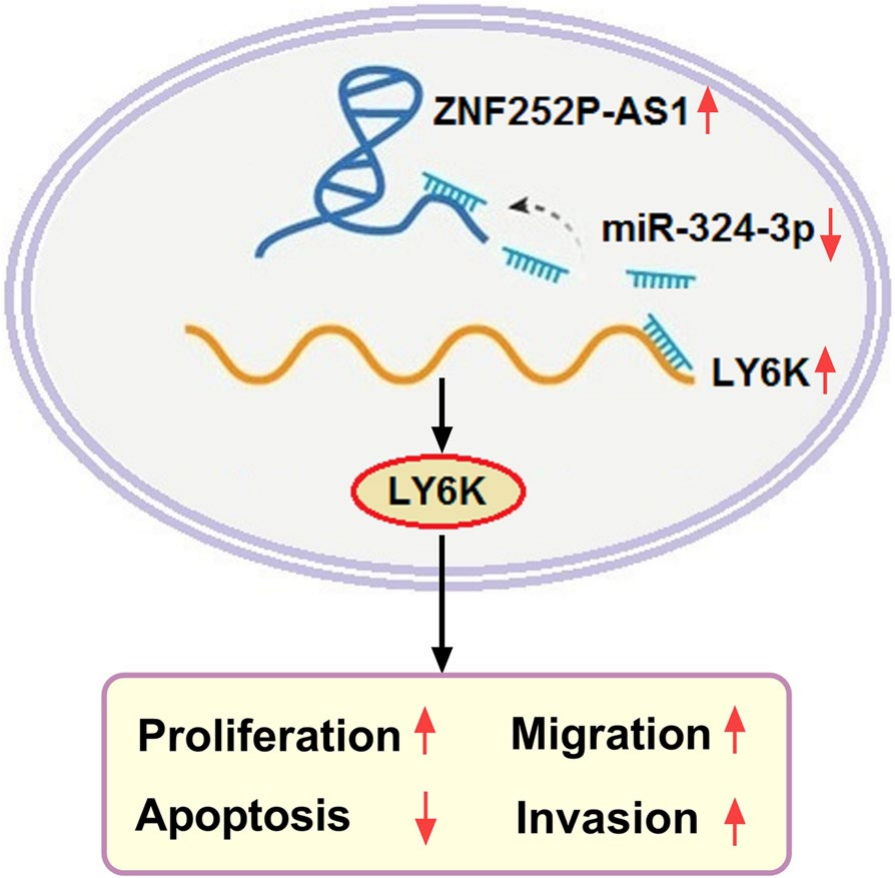
A schematic model of ZNF252P-AS1 functions in ovarian cancer cells. Upregulation of ZNF252P-AS1 enhance LY6K expression by competitively binding miR-324-3p, which promoted the proliferation, migration and invasion, and inhibited the apoptosis of ovarian cancer cells

In this report, we found that ZNF252P-AS1 can competitively bind miR-324-3p to enhance LY6K expression, thereby promoting the proliferation, migration and invasion, and inhibiting the apoptosis of ovarian cancer cells (Fig. 9). ZNF252P-AS1/miR-324-3p/LY6KKnockdown of ZNF252P-AS1 could restrain tumorigenesis of ovarian cancer in mouse models, which suggested that ZNF252P-AS1 might be considered as a new potential therapeutic target for ovarian cancer.

## Data Availability

The datasets used and analyzed during the current study are available from the corresponding author on reasonable request.
